# Success rate to complete optimal 20 + 2 ISUOG planes for foetal ultrasonographic structural screening during early second trimester pregnancy in Thailand

**DOI:** 10.1186/s13089-021-00238-2

**Published:** 2021-08-14

**Authors:** Ravita Chaichanalap, Tharangrut Hanprasertpong

**Affiliations:** Department of Obstetrics and Gynecology, Faculty of Medicine, Srinakharinwirot University, Ongkharak, 26120 Nakornnayok Thailand

**Keywords:** Foetal structural screening, Success rate, Ultrasonographic

## Abstract

**Objective:**

To compare the success rates of obtaining optimal 20 + 2 (2 overview + 20 planes) International Society of Ultrasound in Obstetrics and Gynaecology (ISUOG) planes for foetal ultrasound structural screening between pregnant women at gestational age (GA) 18–20 weeks and 20–22 weeks 6 days

**Methods:**

A prospective descriptive study was conducted. Singleton pregnant women at GA 18–22 weeks + 6 days attending antenatal clinic between December 2019 and March 2020 were invited to participate in the study. Women whose foetuses had obvious structural anomalies were excluded. The ultrasound screening using 20 + 2 ISUOG protocol was performed by 21 operators who had completed the online ISUOG basic training programme with an experience of ultrasound scanning of at least 30 cases. The success rates of achieving optimal planes between GA 18–20 weeks and 20–22 weeks 6 days were compared using Chi-square test. Common suboptimal planes in the ultrasound scan were also presented.

**Results:**

Optimal 20 + 2 ISUOG planes were successfully assessed in 97/126 (77%) and 112/126 (88.9%) patients in the group with a GA < 20 weeks and in the group with a GA ≥ 20 weeks, respectively. Overall success rate was 82.9%. The success rate for the GA < 20 weeks group was significantly lower than that for the GA ≥ 20 weeks group. The group with a GA ≥ 20 weeks had a 1.2 times higher success rate than the group with a GA < 20 weeks. The most common suboptimal planes were the facial planes, especially the median facial profile, and foetal thoracic planes.

**Conclusions:**

We prefer to perform foetal structural screening using US with the 20 + 2 ISUOG protocol at a GA 20 to 22 weeks and 6 days with the aim reducing the need for repeat scans.

## Background

Ultrasonography (US) is a standard investigation for detecting foetal structural abnormalities. There are many reports that have evaluated the detection rate, accuracy, and cost-effectiveness of US in detecting routine foetal structural abnormalities in low-risk pregnancies [1–3]. Most experts suggest performing routine US investigations for foetal structural abnormalities at 18–22 weeks of gestation, although this may be performed earlier to evaluate such abnormalities during the first trimester aneuploidy screening [4]. For a foetal anatomical survey, the evaluation should be systematically examined. International Society of Ultrasound in Obstetrics and Gynaecology (ISUOG) launched the good guidance (the 20 + 2 approach) for obstetricians to perform an effective foetal evaluation since 2018. The protocol consisted of a combination of 2 overview sweeps with 20 planes (divided into 7 anatomical areas: head, thorax, abdomen, pelvis, limbs, spine and face) of the foetus. It also has been backed up by ISUOG online education and training. Each plane relates to a specific foetal section or view that enables the potential exclusion of 50 abnormal foetal appearances.

In Thailand, there is no recommendation about when to perform routine US foetal structural screening. Consequently, the practice varies depending on the location and availability of a suitably qualified physician and US instrument, but US may be performed in certain cases such as genetic Down syndrome screening for high-risk pregnancies [5]. The Antenatal Outpatient Unit, Department of Obstetrics and Gynaecology, Faculty of Medicine, Srinakharinwirot University, serves as a maternal foetal medicine centre for general obstetrics, and for other hospitals in eastern Thailand. We arrange routine US foetal structural screening for all pregnant women who attend the antenatal care (ANC) clinic at our hospital and for pregnant women who visit ANC units at other hospitals or clinics nearby. Generally, we prefer a gestational age (GA) of 18 to 22 weeks and 6 days for screening. Unfortunately, suboptimal screening results are occasionally obtained and lead to the need for a repeat scan during the next visit. A suboptimal screening result and the need for a repeat scan are not ideal for pregnant women or physicians. We postulated that the GA may affect the chance of obtaining suboptimal screening results. Thus, we conducted this study to investigate this hypothesis. The primary objective was to compare the success rates of the complete optimal 20 + 2 (2 overviews + 20 planes) International Society of Ultrasound in Obstetrics and Gynaecology (ISUOG) protocol for foetal US structural screening between women with pregnancies at a GA < 20 weeks and those with a GA ≥ 20 weeks. The secondary objective was to identify the planes that most commonly resulted in suboptimal results. Finally, we also aimed to determine the possible factors that influence the chance of obtaining a suboptimal result.

## Methods

A prospective descriptive study was conducted between December 2019 and March 2020. Singleton pregnant women who visited our ANC clinic during 18 to 22 weeks and 6 days of gestation who were scheduled for routine US foetal structural screening at the Antenatal Outpatient Unit, Department of Obstetrics and Gynaecology, Faculty of Medicine, Srinakharinwirot University, Thailand, were enrolled in our study. The exclusion criteria were refusal to participate or detection of foetal abnormality during US screening.

The study was approved by the institutional ethics committee (SWUEC/E-294/62) and was registered with the Thai Clinical Trials Registry (TCTR 20200107005). After routine ANC was performed, all singleton pregnant women with a GA between 18 and 22 weeks and 6 days were routinely requested to undergo US foetal structural screening. Before the US examination, we explained our study to the women and if they agreed to participate in our study, we asked each woman to give signed informed consent. The participants’ demographic data and possible factors influencing the success rate of the complete optimal 20 + 2 ISUOG protocol were collected, including history of an abdominal surgery. Then, the participants were asked to undergo an US examination performed by the doctor on duty that day. If there was more than one service doctor working that day, the doctor with the greatest availability was asked to do the examination. US screening in our study was performed by 21 operators, classified as 6, 3 or 12 doctors who were qualified to practise maternal foetal medicine by The Royal Thai College of Obstetricians and Gynaecologists (RTCOG), the residents attending of maternal foetal medicine fellowship training programme or obstetrics and gynaecologic training programme, respectively, at the Department of Obstetrics and Gynaecology, Faculty of Medicine, Srinakharinwirot University, under the RTCOG curriculum. The 20 + 2 ISUOG protocol was applied for the screening [6]. We set the qualifications of the operators before taking part in this study, including: (1) completion of the online ISUOG basic training programme, and (2) experience in US scanning of at least 30 cases. For maternal foetal medicine fellowship training doctors, they independently performed US scan without confirmation. For obstetrics and gynaecologic training doctors, all US images were audited by maternal foetal medicine staffs.

All retrieved images of cases and controls were assessed offline by two independent examiners (R.C., T.H.) who were unaware of the final diagnosis.

The US information sought included the 20 + 2 ISUOG foetal anatomical area planes, amniotic fluid deepest vertical pocket (DVP), placental location and thickness, presence of pelvic mass, and maternal anterior abdominal wall thickness. The maternal anterior abdominal wall thickness was measured along the linea nigra at the mid-portion between the umbilicus and the pubic symphysis. The results of the US examination were recorded in the hospital-based system and reported to the primary doctor for antenatal management planning. Each scan took approximately 15 to 20 min. Suboptimal scan defined by operator when the scan took more than 30 min and changing position of pregnant women on the examination bed was tried. If the examination results were suboptimal, the remarks of concerning failure such as foetal position or maternal habitus and suboptimal anatomical area was recorded and the pregnant women were asked to make another appointment for a second scan as per the hospital protocol.

The required sample size was estimated on the basis of sufficiency to compare two independent proportions. Based on a previous study [7], the proportion of successfully completed optimal examinations were 90% and 70% for women with a GA ≥ 20 weeks and GA < 20 weeks, respectively. To achieve a power of at least 80% to detect significant differences at an alpha error of 0.05, beta error of 0.2, and for a sample ratio between two groups of 1:1, the sample size required for each group was determined to be approximately 126 subjects, or 252 in total. The statistical analysis was performed with R2.10.0 software (freeware distributed by the R Development Care Team). The patients’ basic characteristics were collected and the data distribution was analysed by the Kolmogorov–Smirnov test and presented as the number, percentage, median, range, mean, and standard deviation (SD). The differences in the patients’ basic characteristics between the two groups were analysed using the Chi-square test and Fisher’s exact test. The success rates of obtaining complete 20 + 2 ISUOG planes for foetal US screening in each group are presented herein as the number and percentage. The difference in success rates between the two groups was compared using Chi-square test. Other possible factors that may affect the success rate were analysed using the bivariate, co-linearity and multiple logistic regression. Finally, the common foetal structural system when complete 20 + 2 ISUOG planes were successfully obtained for foetal US screening is presented as the number and percentage and was correlated to a GA < 20 weeks or GA ≥ 20 weeks as analysed by Fisher exact test. A probability value of *p* < 0.05 is considered significant in all cases.

## Results

In total, 252 participants were enrolled in the study, with 126 in each group. The patients’ basic characteristics are shown in Table 1. Maternal age, race, body mass index (BMI), gravidity, parity, history of abdominal surgery, type of previous abdominal scar incision, abdominal wall thickness, DVP, placental location, and the presence of myoma uteri showed no statistically significant differences between the two groups. Information about the operator who performed the foetal US screening is presented in Table 2. The median US durations were 15 (12–21), 24 (15–30), and 19 (15–30) minutes for the GA < 20 weeks group, the GA ≥ 20 weeks group, and overall, respectively. The time needed to perform the US was significantly longer for the pregnant women with a GA ≥ 20 weeks than for those with a GA < 20 weeks (*p* < 0.001).

**Table 1:** Patients’ characteristics (*n* = 252)

	GA < 20 weeks (*n* = 126)	GA ≥ 20 weeks (*n* = 126)	Overall (252)	*P*-value
Age (years)				0.892
Median (interquartile range)	29 (23–33)	28.5 (23–32.5)	29 (23–33)	
Race, n (%)				0.593
Thai	106 (49.3%)	109 (50.7%)	215	
Other	20 (54.1%)	17 (45.9%)	37	
BMI (kg/m^2^)				0.656
Median (interquartile range)	23.8 (21.4–26.7)	23.7 (20.8–27.1)	23.8 (21–26.9)	
Gravid, n (%)				0.240
Nulligravida (*G* = 1)	51 (54.8%)	42 (45.2%)	93	
Multigravida (*G* ≥ 2)	75 (47.2%)	84 (52.8%)	159	
Parity, n (%)				0.253
Nulliparous (*P* = 0)	60 (54.1%)	51 (45.9%)	111	
Multiparous (*P* ≥ 1)	66 (46.8%)	75 (53.2%)	141	
History of abdominal surgery, n (%)				0.357
Presence	24 (44.4%)	30 (55.6%)	54	
Absence	102 (51.5%)	96 (48.5%)	198	
Type of previous abdominal scar incision, n (%)				0.694
Midline	5 (50%)	5 (50%)	10	
Transverse	16 (41%)	23 (59%)	39	
Laparoscopic	1 (100%)	0	1	
Other	2 (50%)	2 (50%)	4	
Abdominal wall thickness (cm)				0.889
Median (interquartile range)	1.7 (1.3-2.2)	1.7 (1.3-2.1)	1.7 (1.3-2.1)	
Deep vertical pocket (cm)				0.135
Mean ± SD	3.9 ± 0.9	4.1 ± 1.0	4.0 ± 1.0	
Placental location n (%)				0.130
Anterior	72 (54.5%)	60 (45.5%)	132	
Posterior	54 (45.0%)	66 (55.0%)	120	
Myoma uteri *n* (%)				0.175
Presence	1 (20%)	4 (80%)	5	
Absence	**125 (50.6%)**	**122 (49.4%)**	**247**

**Table 2:** Information about the operator (*n* = 252)

	GA < 20 weeks (*n* = 126)	GA ≥ 20 weeks (*n* = 126)	Overall (252)	*P*-value
Operator, *n* (%)				0.070
Obstetrics and gynaecology residents	17 (37.8%)	28 (62.2%)	45	
MFM fellows	84 (49.7%)	85 (50.3%)	169	
MFM staff with < 5-year experience	14 (60.9%)	9 (39.1%)	23	
MFM staff with ≥ 5-year experience	**11 (73.3%)**	**4 (26.7%)**	**15**	

*MFM* maternal foetal medicine

Overall, 209 of the 252 pregnant women (82.9%) had 20 + 2 ISUOG planes completed for foetal US screening in our study. The success rates were 97/126 (77%) and 112/126 (88.9%) for participants with GA < 20 weeks and GA ≥ 20 weeks, respectively. The success rate for the group with a GA < 20 weeks was significantly lower than the success rate for the group with a GA ≥ 20 weeks. The GA ≥ 20 weeks group had a 1.2 times higher success rate for obtaining complete optimal 20 + 2 ISUOG planes for foetal US screening than the GA < 20 weeks group (Table 3). Other variables, including BMI, history of abdominal surgery, type of previous abdominal scar, presence of myoma uteri, placental location, experience of the operators and maternal abdominal wall thickness, were not statistically significant factors for the success rate of obtaining a complete and optimal scan. The means ± SDs of DVP were 3.98 ± 0.95 cm and 3.95 ± 0.99 cm in the optimal scan and suboptimal scan groups, respectively, which was not a significant difference (*p* = 0.87). The median (interquartile range) maternal abdominal wall thicknesses were 1.66 (1.34–2.10) cm and 1.78 (1.31–2.56) cm in the optimal scan and suboptimal scan groups, respectively, which was not a significant difference (*p* = 0.39). Subgroup analysis of US screening performed by maternal foetal medicine fellows showed that the suboptimal rate of the GA < 20 weeks group was 3 times significantly higher than that of GA ≥ 20 weeks group (25.0% VS 8.2%, p-value = 0.003).

**Table 3:** Factors affecting the chance of success of obtaining complete 2 + 20 ISUOG planes for foetal ultrasonographic screening

Variables	Result		Prevalence rate ratio	95% CI	*P*-value*
	Incomplete	Complete			
GA					0.012
> 20 weeks (%)	14 (11.1)	112 (88.9)	1.2	1.031–1.294	
< 20 weeks (%)	29 (23)	97 (77)			
BMI					
< 25 kg/m^2^ (%)	22 (14.2)	133 (85.8)	1.1	0.969–1.238	0.126
> 25 kg/m^2^ (%)	21 (21.6)	6 (78.4)			
History of abdominal scar			1.1	0.974–1.227	0.190
Yes (%)	6 (11.1)	48 (88.9)			
No (%)	37 (18.7)	161 (81.3)			
Myoma uteri			1.2	1.143–1.282	0.306
Yes (%)	0	5 (100)			
No (%)	43 (17.4)	204 (82.6)			
Placenta			1.0	0.920–1.152	0.609
Anterior (%)	21 (15.9)	111 (84.1)			
Posterior (%)	22 (18.3)	98 (81.7)			
Presentation			1.0	0.912–1.144	0.716
Non-cephalic (%)	16 (16)	84 (84)			
Cephalic (%)	27 (17.8)	125 (82.2)			
Sonographer					
Staff (%)	10 (26.3)	28 (73.7)	0.871	0.714-1.062	0.100
Resident and fellow (%)	33 (15.4)	181 (84.6)			

*The univariate, co-linearity and multiple logistic regression analysis

Table 4 presents the most common foetal suboptimal planes, which were the facial (especially median facial profile) and foetal thoracic planes. The common failure in foetal thoracic planes was presented. The most common suboptimal planes were not significantly different between the two groups. All suboptimal examinations were optimal scan in the next subsequent appointment. No structural anomalies were reported in postnatal examination by routine neonatal evaluation in all participants. The common reasons for suboptimal scan were foetal position (86%) and maternal habitus (9.3%). The detail of foetal position did not record in our study.

**Table 4:** Details about the foetal suboptimal scanning plane

Suboptimal planes	GA < 20 weeks (*N* = 126)	GA > = 20 weeks (*N* = 126)	Overall (*N* = 256)	*P* value
Face (*n*, %)	13 (10.3)	9 (7.1)	22 (8.6)	0.240
Coronal view if upper lip, nose and nostrils (n. %)	3	0	3	
Both orbits,,both lens (n, %)	1	0	1	
Median facial profile (n, %)	9	9	9	
Thorax (*n*, %)	4 (3.2)	4 (3.2)	8 (3.1)	0.086
Lung, 4CB (n, %)	0	0	0	
LVOT (n, %)	0	0	0	
RVOT and crossover of LVOT (*n*, %)	1	0	1	
3VT (*n*, %)	1	0	1	
RVOT and crossover of LVOT and 3VT (*n*, %)	0	1	1	
LVOT, RVOT and crossover of LVOT and 3VT (*n*, %)	2	0	2	
Lungs, 4CB, LVOT, RVOT and crossover of LVOT and 3VT (*n*, %)	0	3	3	
Face and thorax (*n*, %)	11 (8.7)	1 (0.8)	12 (4.7)	
Face and abdomen (*n*, %)	1 (0.8)	0 (0)	1 (0.4)	

*4CB* 4 chamber view of heart, *LVOT* Left ventricular outflow tract, *RVOT* Right ventricular outflow tract, *3VT* 3 vessel trachea view of heart

^*^Fisher exact test

## Discussion

Our present study is the first to evaluate the success rate of obtaining complete and optimal 20 + 2 ISUOG planes for foetal US structural screening during the early second trimester in a developing country, namely Thailand. In low-resource developing countries, the number of qualified and trained US physicians and the availability of US equipment is limited compared to the number of pregnant women. Thus, effective medical resource management is necessary. Our study chose to implement the 20 + 2 ISUOG protocol because foetal structural US screening using such a standard protocol improves the sensitivity of screening for all anomalies and major anomalies in populations of varying risk [8]. The application of a standard protocol is an advantage of our study. Moreover, attending the ISUOG basic training online course improves the practitioners’ theoretical knowledge and practical skills [9]. Thus, all physicians should attend such a course before performing US.

The overall success rate of obtaining optimal 20 + 2 ISUOG planes in our study was 82.9%. The success rates were 97/126 (77%) and 112/126 (88.9%) for participants with a GA < 20 weeks and GA ≥ 20 weeks, respectively. In previous studies, the success rates were reported to be approximately 86–94% when the examinations were performed during a GA = 18–20 weeks [10–13]. The success rate in our study is slightly lower than that in previous studies. We hypothesized that the lower rate resulted from the variation in physician experience. All previous studies concerned developed countries, where US examinations are performed by maternal foetal medicine-specialized obstetricians, while the examinations in our present study were performed by physicians with different experience levels, including maternal foetal medicine practitioners who had qualified as doctors of obstetrics or maternal foetal medicine fellowship-residents and residents in training. However, the success rates among operators with similar experience level were not different either in GA ≥ 20 weeks or GA < 20 weeks groups. Regarding the success rate of obtaining optimal 20 + 2 ISUOG planes between pregnant women with a GA ≥ 20 weeks and GA < 20 weeks, we found that those with a GA ≥ 20 weeks had a 1.2 times higher success rate for foetal US screening than women with a GA < 20 weeks. This is similar to previous studies, which found that a lower GA decreases the success rate of achieving optimal visualization [11–13]. We postulated that the smaller foetus and relatively smaller foetal size to amniotic fluid ratio may affect the possibility of achieving success, and hence the success rate. Moreover, the operator’s confidence in making a conclusion about a normal foetal finding may be decreased when the US is performed for women with a lower GA. Furthermore, the confidence of the operator may be more affected in procedures performed by a less experienced doctor than in those performed by a more highly experienced doctor. Importantly, there is a greater risk that a diagnosis or suspicion of some malformations may be reported as a false positive when the assessment is performed in early pregnancy, such as the case for mild pyelectasis [13].

In addition to a GA ≥ 20 weeks or GA < 20 weeks, other factors did not significantly affect the success rate of obtaining optimal 20 + 2 ISUOG planes, including BMI, history of abdominal surgery, abdominal wall thickness, DVP, placental location, and the presence of myoma uteri. This finding is different from a previous study, which reported that foetal position, oligohydramnios, prior abdominal surgery, and maternal obesity may account for suboptimal US visualization. We hypothesized that our different result may be caused by the definition of each variable, number of sample size calculation which based on GA only and small proportion of some maternal characters such as presence of myoma, obese or abdominal scar, etc [11, 14–16]. The most common suboptimal planes in our study were the facial profile and thoracic planes. These results were similar to those another previous study [17]. We hypothesized that the success of getting both facial profile and thoracic planes depends on foetal position. Finally, the limitation of the study should be addressed. We excluded foetuses with obvious anomaly and evaluated only the success rates of achieving optimal planes of ultrasound scanning in normal foetuses. Therefore, a diagnostic performance of the 20 + 2 ISUOG protocol to detect foetal anomalies could not be assessed. Future research should be performed focusing on this issue. Re-schedule for US scan on the same day and waiting for foetal position changing may be optioned.

## Conclusion

Based on the results of our study, we prefer to perform foetal structural screening using US with the 20 + 2 ISUOG protocol at a GA 20 to 22 weeks and 6 days with the aim of reducing the chance that the patient will need a repeat scan. We recommend this as a preferred foetal US structural screening practice during the early second trimester of pregnancy in Thailand, and in other developing countries (resource-limited areas). Anyway, delaying foetal abnormal diagnosis should be cautioned.

## Data Availability

The datasets used and/or analysed during the current study are available from the corresponding authors on reasonable request.
